# Parallel evolution despite low genetic diversity in three-spined sticklebacks

**DOI:** 10.1098/rspb.2023.2617

**Published:** 2024-04-10

**Authors:** Carla Coll-Costa, Carolin Dahms, Petri Kemppainen, Carlos M. Alexandre, Filipe Ribeiro, Davor Zanella, Linda Zanella, Juha Merilä, Paolo Momigliano

**Affiliations:** ^1^ Ecological Genetics Research Unit, Organismal and Evolutionary Biology Research Programme, Faculty of Biological and Environmental Sciences, University of Helsinki, Helsinki, FI-00014, Finland; ^2^ School of Biological Sciences, Faculty of Science, The University of Hong Kong, Hong Kong SAR, People's Republic of China; ^3^ Swire Institute of Marine Science, Faculty of Science, The University of Hong Kong, Hong Kong SAR, People's Republic of China; ^4^ MARE—Marine and Environmental Sciences Centre, Universidade de Évora, Évora, 7004-516, Portugal; ^5^ MARE—Marine and Environmental Sciences Centre, Faculdade de Ciências, Universidade de Lisboa, Campo Grande, 1749-016, Lisboa, Portugal; ^6^ Department of Biology, Faculty of Science, University of Zagreb, Rooseveltov trg 6, Zagreb, 10000, Croatia

**Keywords:** adaptation, *Gasterosteus aculeatus*, genetic diversity, parallel evolution

## Abstract

When populations repeatedly adapt to similar environments they can evolve similar phenotypes based on shared genetic mechanisms (parallel evolution). The likelihood of parallel evolution is affected by demographic history, as it depends on the standing genetic variation of the source population. The three-spined stickleback (*Gasterosteus aculeatus*) repeatedly colonized and adapted to brackish and freshwater. Most parallel evolution studies in *G. aculeatus* were conducted at high latitudes, where freshwater populations maintain connectivity to the source marine populations. Here, we analysed southern and northern European marine and freshwater populations to test two hypotheses. First, that southern European freshwater populations (which currently lack connection to marine populations) lost genetic diversity due to bottlenecks and inbreeding compared to their northern counterparts. Second, that the degree of genetic parallelism is higher among northern than southern European freshwater populations, as the latter have been subjected to strong drift due to isolation. The results show that southern populations exhibit lower genetic diversity but a higher degree of genetic parallelism than northern populations. Hence, they confirm the hypothesis that southern populations have lost genetic diversity, but this loss probably happened after they had already adapted to freshwater conditions, explaining the high degree of genetic parallelism in the south.

## 1. Introduction

Parallel evolution, defined as the evolution of similar phenotypes based on shared genetic mechanisms [1–3], takes place when different species or populations repeatedly adapt to similar environments. Studying parallel evolution is central to understanding local adaptation, as repeated phenotypic changes within similar environments directly imply the role of natural selection in the evolution of these traits [4]. Theoretical [5], experimental [6] and empirical [7,8] studies have demonstrated that selection on adaptive alleles which are identical by descent and harboured as standing genetic variation (SGV) in the source population is one of the most important pathways to rapid parallel evolution. Indeed, many of the most spectacular examples of rapid parallel evolution of ecotypes within species can be explained by selection on SGV whose origin is far more ancient than the newly colonized populations [9–12].

The likelihood of parallel evolution from SGV is influenced by the effective population size (*N_e_*) of both the source population and the populations invading the new environment [5,13,14], gene flow [15,16], and is expected to be higher for large- than small-effect-loci [17]. This is because in small populations potentially beneficial alleles are more likely to be stochastically lost, and the effects of genetic drift [18,19] or migration [20] may override the effects of natural selection, lowering the probability of adaptation, particularly for loci of small effect [16,21]. Understanding how demographic factors affect the likelihood of parallel evolution is also important to understand the evolutionary potential of populations. Both processes are limited by available SGV and the same demographic factors that reduce the likelihood of parallel evolution restrict the potential for local adaptation [16].

The three-spined stickleback (*Gasterosteus aculeatus* L.) is a model organism for studies of local adaptation, especially for the study of parallel evolution [15,22–24]. The contemporary species range extends across most of the northern hemisphere [25–27]. Three-spined sticklebacks originated in the northern Pacific [28–30], from where they colonized the Atlantic Ocean and then the Baltic Sea, the Mediterranean Sea and the Black Sea [30]. Within all these different biogeographic regions, marine stickleback populations successfully invaded different brackish and freshwater habitats, evolving similar morphological traits as adaptations to life in low salinity environments [26,31–33]. These adaptations involve several traits, such as body armour and lateral-plate reduction in freshwater populations [34], male courtship behaviour [35], body shape, physiological adaptations to different salinities and trophic specialization [31].

The high prevalence of multi-trait parallel evolution in this species has been attributed to the mechanism described by the ‘transporter hypothesis' [1]. The hypothesis posits that adaptive alleles in freshwater populations are transported to marine populations, where they are maintained at low frequencies via recurrent gene flow. Recombination in the marine population weakens the correlation among freshwater-adapted alleles, disintegrating the freshwater haplotype. As new streams are formed and colonized from the sea, selection and recombination reassemble the freshwater-adapted haplotype in the newly colonized location, allowing rapid responses to selection on ecological timescales [12]. Recent studies, however, demonstrated that this parallelism is not universal and is constrained by demographic history, especially by effective population size (*N*_e_) and gene flow [3,16,36–39]. Most notably, it has been shown that signatures of parallel evolution in this species are strongest in the Eastern Pacific region and lower in other parts of the world [15,37,38].

During Pleistocene glaciations, three-spined stickleback populations inhabiting high-latitude areas of Europe were eradicated, whereas populations residing in (or moving to) the south persisted in refugia [27]. After the retreat of the ice sheets covering northern Europe, the high-latitude areas became recolonized by migration from the south, and hence, today's northern European populations are relatively young [27,30,38,40,41]. These contrasting histories between high and low latitude populations explain some of the clear differences we see today in both genetic diversity and differentiation among populations from these latitudinal extremes.

Population genetic studies of *G. aculeatus* have been focused on high-latitude areas, where freshwater populations are typically less than 10 000 years old. Few studies have focused on lower-latitude areas [27,40,42,43], and these have used a limited number of microsatellite markers and mtDNA. As genetic diversity usually decreases moving away from the centre of origin of a range expansion [44], there are good reasons to believe that southern populations (some of which may be over 40 000 years old [30,41,45]) host most of the ancestral polymorphism that fuelled subsequent expansions to the north. Indeed, this pattern is observed in many European species that had glacial refugia in Southern Europe [46,47]. However, recent work on sticklebacks from the Adriatic Sea area revealed lower genetic diversity than in northern Europe [45].

Nowadays European marine three-spined stickleback populations extend from the Bay of Biscay through the north up to the Barents Sea and can be also be found in the Black Sea, but remain absent from the Iberian Atlantic coast and the Mediterranean Sea, with the exception of certain coastal lagoon areas [48]. On the other hand, freshwater populations have a continuous distribution from Mediterranean coastal areas up to the Barents Sea [25,32,42]. Consequently, current freshwater populations from southern Europe are unlikely to be in contact with the marine source population(s). This has likely subjected the southern European populations to stronger genetic drift, and erased part of the ancestral polymorphism. Indeed, southern European freshwater populations show a high degree of genetic differentiation over very short geographic distances [42,49].

This study had two main aims. First, to investigate whether the southern European freshwater populations (mainly in the Iberian Peninsula and around the Mediterranean basin) of the three-spined stickleback—which currently lack or have limited connection to marine populations—have lost genetic diversity due to population bottlenecks and inbreeding compared to their northern European counterparts (populations from Fennoscandia). Second, to compare the extent of parallel evolution in southern versus northern European freshwater populations. To this aim we focused on specific genomic regions which have been shown to be consistently associated with freshwater colonization across a very broad geographic range in earlier studies [15,23] and display high linkage disequilibrium (LD) [15]. Under the assumption that the lack of continued access for freshwater populations to SGV in the ancestral marine population reduces the likelihood of parallel evolution, we hypothesized that the degree of genetic parallelism (here defined as the proportion of individuals exhibiting freshwater adapted haplotypes) in genomic regions subject to positive selection in freshwater environments is lower in southern than in northern European populations. If, however, a reduction in genetic diversity and/or cessation of gene flow between southern European freshwater and marine populations occurred following freshwater adaptation, we expected a lower degree of genetic parallelism in northern than southern European populations.

## 2. Methods

### (a) Sampling design and sample collection

**Figure 1.**
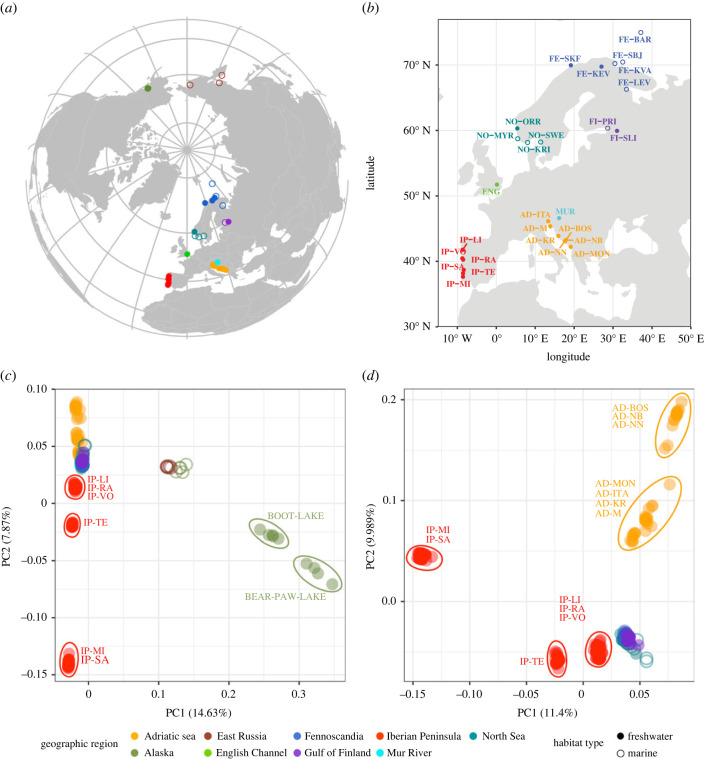
Location of samples and PCA (*a*) World map showing the location of the populations analysed and their habitat type. (*b*) Location of European samples. (*c*) PCA performed using genome-wide SNPs of all samples clusters all European populations together and shows two clusters in the Pacific, one corresponding to marine and the other to freshwater populations. (*d*) PCA of European samples shows three clusters for Iberian Peninsula populations, one cluster for all northern European populations and one large cluster that could be divided into two for the Adriatic Sea populations. Samples are coloured according to geographical region. Filled and empty circles represent freshwater and marine populations, respectively.

In total, the sampling design included 257 individuals from 36 populations (figure 1).

We obtained 149 samples from 11 populations from the Adriatic Sea basin and the Iberian Peninsula between 2003 and 2015. All samples were collected during the local breeding seasons using mist-nets, minnow traps or electric fishing, under appropriate permits (see Ethics).

We also retrieved previously published RAD-seq data from an additional 118 individuals: 14 previously sequenced individuals collected from marine and freshwater Alaskan populations from Nelson and Cresko [12], 27 individuals from several European locations previously sequenced by Fang *et al.* [30], and 67 individuals from European and eastern Russian locations sequenced by Fang *et al.* [3]. To study genetic parallelism, samples were classified as belonging to one of the following biogeographic regions [30]: Adriatic Sea, Alaska, Eastern Russia, English Channel, Fennoscandia, Gulf of Finland, Iberian Peninsula, Mur River and North Sea. The Mur River population is geographically close to the Adriatic Sea populations, but it was classified in a different biogeographic region because it belongs to the Danube Basin. Details of samples with their respective population names, geographic coordinates, country, habitat type, sampling years, DNA extraction methods and source of the sequence information are given in electronic supplementary material, tables S1 and S3.

### (b) Laboratory methods

#### (i) Sequencing

Genomic DNA was extracted from alcohol-preserved samples using different methods (see electronic supplementary material, table S1), and extracted DNA was shipped to BGI Hong Kong for library preparation. Library preparation and sequencing details for Alaskan samples can be found in Nelson & Cresko [12]. For all other samples the methods were identical to those in Fang *et al*. [30]. Briefly, restriction site associated DNA sequencing (RAD-seq) [50,51] was performed using the PstI enzyme and a P1 adaptor with unique barcodes. Forward amplification and sequencing primers were ligated. Ligation products were randomly sheared, and size selected using a 300–500 bp window. P2 adaptors were ligated and fragments with P1 and P2 were selectively amplified by PCR. The libraries were sequenced on an Illumina HiSeq2000 platform (150 bp paired-end sequencing).

#### (ii) Data preparation

PCR duplicates were removed with the *clone_filter* program, part of the Stacks pipeline [52]. The remaining read pairs were mapped to the Broad S1 reference genome (release 101), retrieved from the Ensembl database [53], using *BWA* *v.0.7.17* [54] and *SAMtools v.1.10* [55]. Given that we observed some heterogeneity in sequencing depth among samples, we performed most analyses using genotype likelihoods, although some analyses required hard-called genotypes. Variants were called using *GATK v.4.0.1.2* following GATK best practices workflow [56]. A gVCF was created for each individual using *HaplotypeCaller*, all gVFCs were combined using *CombinegVCFs*, and a VCF with genotype likelihoods and genotype calls was produced using *GenotypeVCF*. The VCF was filtered to retain only biallelic SNPs with less than 25% missing data and a genotype quality above 20, resulting in a dataset of 16 040 046 SNPs. For a detailed description of the data analysis pipeline, please refer to the annotated scripts deposited in GitHub (https://github.com/Nopaoli/3s-Parallel-Evolution) and in Dryad [57].

#### (iii) Genetic diversity

We estimated observed individual heterozygosity (*H_O_*) and nucleotide diversity (π) [58] from individual and population site frequency spectra generated with *ANGSD*, using the scripts from Momigliano *et al.* [59]. We analysed runs of homozygosity (ROH) [60], long stretches of homozygous genotypes arising at a locus owing to identity-by-descent, to provide insight on the demographic history of the sampled populations [61]. We calculated ROH from the input VCF file, further filtered to retain only loci with <10% missing data and a minor allele frequency (MAF) above 0.01 (resulting in 138 972 SNPs), using genotype likelihoods (PL) and plotted the number of ROH (*N_ROH_*) against the sum of ROH (*S*_ROH_) for each individual. *F*_ROH_, defined as the proportion of the autosomal genome covered by ROH, was calculated using ROH of all size classes, so no length cut-off was applied. ROH reflect population size, as small populations have more and larger ROH than larger ones (high *N*_ROH_ and *S*_ROH_). Consanguinity, on the other hand, generally results in less numerous but longer ROH [61].

We tested the correlation between habitat type and latitude with genetic diversity using linear models where both predictors were fitted to π and mean *F*_ROH_. The intraclass correlation coefficient (ICC = 0.45 < 0.5) indicated there was no strong correlation between the predictors. In addition, a one-way ANOVA was performed to test for significant differences in π across geographic regions. Geographic locations with less than two observations were removed (English Channel). A *post-hoc* analysis with Tukey-Kramer test was performed to check which pairs of geographic locations showed significant differences in π. For all analyses diagnostic plots were examined to check that the assumptions of normality of residuals and homoscedasticity were met.

#### (iv) Population structure

Pairwise *F_ST_* estimates were obtained with the R package *diveRsity* [62]. For this we used the same data as per ROH analysis, but thinned so that adjacent SNPs were no closer than 10 000 bp, reducing the number of sites from 138 972 to 27 614. Populations with fewer than two individuals after filtering (ENG, NO-KRI, NO-SWE, AD-ITA and FE-BAR) were removed.

A PCA using genome-wide SNPs was performed using *plink v.1.90* (Purcell *et al.* [63]), excluding individuals with average depth <2, retaining only bi-allelic SNPs with genotype quality above 20 and <25% of missing data. This filtering reduced the dataset from 257 to 230 individuals (electronic supplementary material, table S2) and from 16 040 046 to 1 492 678 sites.

#### (v) Demographic history

We estimated one dimensional folded site frequency spectra using *ANGSD* (using the parameter flags *-uniqueOnly 1 -remove_bads 1 -minMapQ 20 -minQ 20 -C 50*), and reconstructed changes in *N*_e_ in southern and northern European populations using *stairwayplot2* [64] using the same parameters as Dahms *et al.* [45]. Regions including chromosomal inversions as well as all LD clusters involved in parallel evolution were removed.

#### (vi) Genetic parallelism

To study the degree of genetic parallelism among the stickleback populations we focused on twelve LD clusters (i.e. groups of loci forming high LD networks) [65] associated with freshwater adaptation across the Atlantic and Pacific regions [3] (LD clusters 5, 6, 10, 11, 12, 13, 16, 18, 20, 22, 25 and 27), which cover 58% of the regions involved in parallel evolution identified by Jones *et al.* [23]. A principal component analysis (PCA) based on loci for each of these LD clusters is expected to separate individuals showing the marine and freshwater adapted genotypes along the 1st PC [3], as for these LD clusters the freshwater adapted alleles are nearly fixed in both Eastern Pacific and Eastern Russia freshwater populations. Therefore, we can use a quadratic discriminant analysis based on the computed PCs trained using Eastern Russian and Alaskan populations to assign, for each LD Cluster, the rest of individuals to a ‘freshwater’ or ‘marine’ haplotype.

Before the analyses we split two clusters that spanned large non-contiguous regions (clusters 11 and 27) into five smaller contiguous clusters (Clusters 11a, b, c and Clusters 27a and b). Furthermore, as patterns of LD can be distinct in different geographic regions, we ran linkage disequilibrium network analyses (LDna) [65] to retain, within each cluster, only the group of loci showing the strongest parallel changes in allele frequencies across marine-freshwater habitats (see relevant section in the electronic supplementary material, figure S1). Information on the chromosome, number of loci and genomic coordinates of each LD cluster within the Broad S1 reference genome (release-101) are given in electronic supplementary material, table S4.

We then performed PCAs for each cluster using two different methods. For the first method, for each LD cluster we estimated the covariance matrix directly from genotype likelihoods using *PCAngsd* [66], filtering for minimum allele frequency of 0.05. We then imported the covariance matrices in R using the *RcppCNPy* library [67] and calculated eigenvalues and eigenvectors.

For the second method, we performed a PCA from hard-called data with the Pacific samples (marine populations: RABBIT-SLOUGH, RUS-AN, RUS-ASH, RUS-KHA, freshwater populations: BEAR-PAW-LAKE, BOOT-LAKE) and projected the European samples on the PCA space. This method was employed to ensure that southern European populations, which are the most abundant in the dataset, do not exert a stronger influence on the PCA space than other samples. The hard-called data were generated by firstly calculating the individual allele frequencies and posterior genotype probabilities from the beagle files for each LD cluster using *PCAngsd*. Second, genotypes were called from posterior genotype probabilities incorporating the individual allele frequencies as prior information. When performing the PCA using the Pacific samples, we removed loci with >20% missing data and replaced remaining missing data with the population mean. To project the European samples on the PCA space we removed the loci that had been removed in the Pacific samples and replaced the NA values following the same criteria as previously described.

For each PCA approach, we used a quadratic discriminant analysis (QDA, implemented in the R package *MASS* [68]) on the principal components (PCs) of each LD cluster to classify individual genotypes as marine or freshwater adapted [68]. Since the degree of genetic parallelism is highest in Pacific populations of three-spined stickleback and we studied LD clusters known to be associated with genetic parallelism at the global scale, we used our Pacific populations (marine populations: RABBIT-SLOUGH, RUS-AN, RUS-ASH and RUS-KHA, freshwater populations: BEAR-PAW-LAKE and BOOT-LAKE) as a training set to classify European individuals. For each LD cluster we built four models, each one adding a new PC: in the first model we tested the PC1, in the second model the PC1 + PC2, in the third model the PC1 + PC2 + PC3 and in the fourth PC1 + PC2 + PC3 + PC4. We determined the accuracy of each of the models using leave-one-out cross-validation on the training dataset, and retained clusters with an accuracy>0.75. After determining the accuracy of the models, we plotted the results of the model with the highest accuracy for each LD cluster. The resulting test gives for each individual and LD cluster an assignment score ranging from 0 (marine genotype) to 1 (freshwater genotype), which we refer to as the Probability of carrying the Freshwater Genotype (PFG). It is important to keep in mind the limitations of the QDA approach that was followed. First, because Pacific populations were used as a training set, what is being specifically compared is the similarity to Pacific haplotypes. Second, the results are limited to regions that are considered globally parallel and may not reflect local parallelism within the European context. The possibility of identifying genomic regions of parallelism unique to the European populations was precluded by the SNP data resolution of our samples and the absence of marine counterparts in the south.

Finally, we generated a parallelism index (PI) for each population. For each LD cluster, we generated a PI by taking the average PFG across individuals (a measure of the proportion of individuals that have the freshwater-adapted haplotype). We then estimated the mean PI for each population across all LD clusters. We used a one-way ANOVA to test for differences in PI across the three European regions: Northern Europe, Iberian Peninsula and Adriatic, and used a Tukey's honest significance test for pairwise comparisons. It's important to note that the (PI) of a population as measured using PFG might represent different genomic regions across individuals.

## 3. Results

### (a) Population structure

The pairwise *F*_ST_ matrix showed clear geographic patterns of genetic differentiation (electronic supplementary material, figure S2). In the Pacific Ocean region, marine populations (RABBIT-SLOUGH, RUS-AN, RUS-ASH, RUS-KHA) have low levels of genetic differentiation, while freshwater populations showed higher levels of differentiation (electronic supplementary material, figure S2). In Northern Europe populations showed low pair-wise levels of differentiation independently of the habitat type (electronic supplementary material, figure S2). Within the Adriatic Sea and Iberian Peninsula populations showed the highest levels of pairwise genetic differentiation (electronic supplementary material, figure S2).

The PCA identified three clusters along the 1st PC axis: a European cluster, a Pacific marine cluster (RABBIT-SLOUGH, RUS-AN, RUS-ASH and RUS-KHA) and a Pacific freshwater cluster (BEAR-PAW-LAKE and BOOT-LAKE; figure 1*c*). Analysis excluding the Pacific samples distinguished six less divergent clusters: three Iberian Peninsula clusters, two Adriatic Sea clusters and a northern European cluster (figure 1*d*). The most separated cluster along the 1^st^ PC axis consisted of the Iberian Peninsula populations IP-MI and IP-SA (figure 1*d*). One other cluster is exclusively formed by the IP-TE Iberian Peninsula population and another cluster by the rest of the Iberian Peninsula populations (IP-LI, IP-RA, IP-VO). The 2nd PC separated the Adriatic Sea and Northern European populations (figure 1*c*). The Adriatic Sea clusters that could be distinguished were formed by AD-MON, AD-KR, AD-ITA and AD-M populations and by AD-BOS, AD-NB and AD-NN populations (figure 1*d*). These latter populations are geographically proximate, while the former ones are genetically differentiated from each other.

### (b) Demographic history

Most freshwater populations from southern Europe showed marked declines in *N*e within the past 10 000 years, suggestive of either a recent colonisation of freshwater habitats or a recent cessation of gene flow with marine populations (electronic supplementary material, figure S4). A few showed evidence of strong bottlenecks followed by population growth (electronic supplementary material, figure S4).

### (c) Genetic diversity

#### (i) ROH analyses

**Figure 2.**
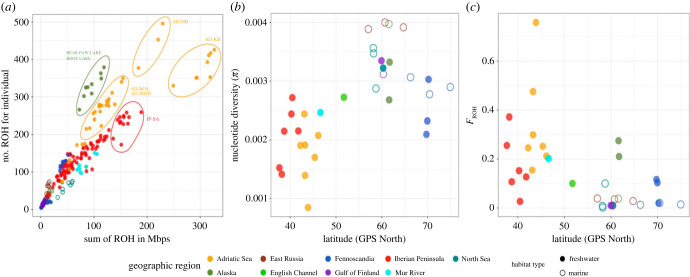
*N*_ROH_ versus *S*_ROH_ and nucleotide diversity and mean *F*_ROH_ as a function of latitude. (*a*) The distribution of *N*_ROH_ against *S*_ROH_ shows that the Adriatic Sea, Iberian Peninsula and Pacific populations have experienced demographic phenomena that have increased the homozygosity. (*b*) Mean nucleotide diversity and (*c*) mean *F*_ROH_ as a function of latitude. Samples are coloured according to the geographic clusters used for the analyses. Filled and empty circles represent freshwater and marine populations, respectively.

Marine individuals had low values of both *N_ROH_* and *S_ROH_*, whereas most freshwater individuals tended to have higher *N*_ROH_ and *S*_ROH_ values (figure 2*a*). The highest *N_ROH_* and *S_ROH_* values were found from the Adriatic Sea (KR in particular), Iberian and Alaskan freshwater populations (figure 2*a*).

#### (ii) Correlation between habitat type and latitude with genetic diversity

Latitude and habitat type had a significant effect on π (Latitude: *F*_1,30_ = 24.978, *p* = 2.344 × 10^−05^; Habitat: *F*_1,30_ = 10.76, *p* = 0.0026; table 1) and the model explained more than 50% of the variation in π (adj-*R*^2^ = 0.5132). Latitude (*F*_1,30_ = 23.053, *p* = 4.08 × 10^−05^) and habitat type (*F*_1,30_ = 15.94, *p* = 0.00039) had significant effects on *F_ROH_* (table 1) and the model, constructed using the gamma family distribution and a log link function, explained more than 25% of the variation in mean *F*_ROH_ (adj-*R*^2^ = 0.2553). Hence, π was higher in marine than in freshwater populations and increased with latitude whereas *F_ROH_* showed the opposite patterns (figure 2*b,c*). An ANOVA showed that π differed significantly among different geographic regions (*F*_7,24_ = 11.6, *p* = 2.32 × 10^−06^), and post-hoc analyses indicated that the Adriatic Sea populations had lower π compared to other regions (except for Iberian Peninsula and Mur River populations, electronic supplementary material, figure S3). Among northern populations, the only significant difference in π was found between Eastern Russia and Fennoscandian populations, the latter having lower π (electronic supplementary material, figure S3).

**Table 1.** Correlation between habitat type and latitude with genetic diversity estimates at population level. Given are the regression coefficients and associated *p* values.

model	response variable	predictor	estimate	*p*
linear model	nucleotide diversity	intercept	1.015 × 10^−03^	0.0666
		latitude	2.513 × 10^−05^	0.0223
		habitat type	8.19 × 10^−04^	0.0026
linear model gamma distribution log link	*F*_ROH_	intercept	0.2671	0.7249
		latitude	−0.0386	0.0134
		habitat type	−1.4433	0.0003

### (d) Genetic parallelism

**Figure 3.**
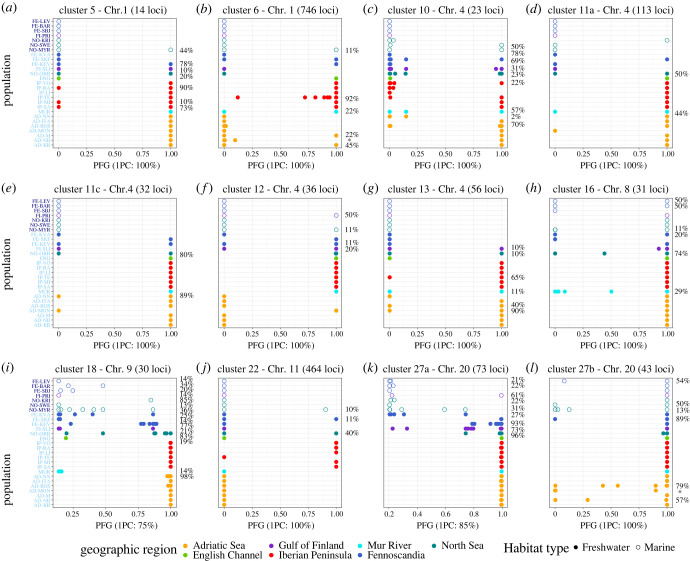
Quadratic discriminant analysis (QDA) of LD-clusters of global parallelism, based on projected PCs. QDA showing the model with the best accuracy for each of the LD clusters of global parallelism. The accuracy and the number of PCs used for the models in each of the clusters can be seen in the x axis. Percentages show the proportion of individuals from each population that are predicted to have the freshwater genotype. For the populations with no percentage, PGF approaches either 0 or 1 for all individuals. Samples are coloured according to geographic location and shaped according to habitat type. Dark blue labels represent marine populations, sky blue labels freshwater populations.

**Figure 4.**
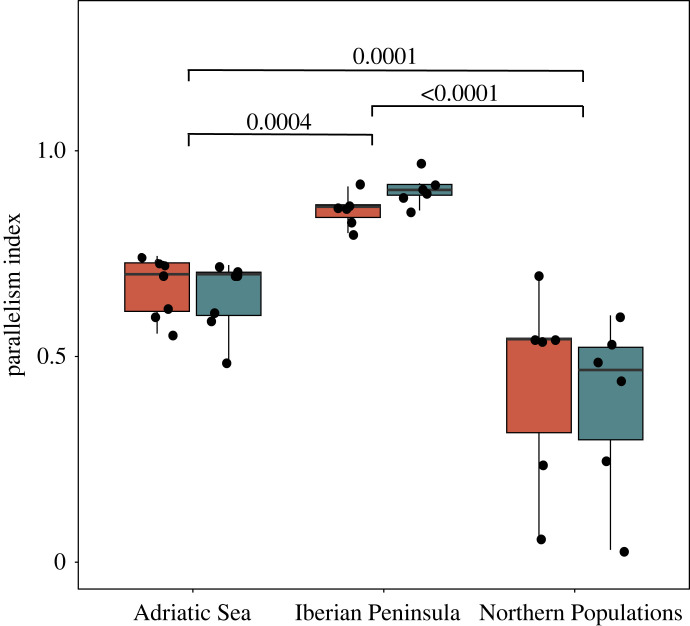
Differences in Parallelism Index (PI) between regions. Each point is the average PI across LD clusters for each population. p values from Tuckey's test show significant differences between PI means. Red boxplots represent the PI derived from QDA based on non-projected PCs, blue boxplots represent the PI derived from QDA based on projected PCs.

The results from both the projected (figure 3) and non-projected (electronic supplementary material, figure S5) QDA show that most of the LD clusters present high accuracy of the model independently of the approach. Clusters 25 was removed from analyses as accuracy estimated from cross-validation was <0.75. Clusters 11b and 20 were removed only from the projected QDA analyses, as they showed low accuracy in the QDA from projected data but not for the unprojected. For most of the clusters, southern European freshwater individuals had PFGs approaching 1 more often than individuals from the northern European freshwater populations (figure 3 and electronic supplementary material, figure S5), with Iberian Peninsula populations showing the strongest patterns of parallelism. These patterns are also clearly apparent from the PC1 scores obtained from PCA from the projected and non-projected PCAs (electronic supplementary material, figures S6 and S7). The Parallelism Indices differed significantly among regions, with freshwater populations from the Iberian Peninsula having the highest and the Northern European populations having the lowest (figure 4; ANOVA: *F*_2,35_ = 35.78, *p* < 0.001)

Visualising PCAs (cores of the 1st PC) of the LD clusters of global parallelism shows a similar patterns for both approaches, with marine populations grouping together as expected, and a tendency for a more pronounced grouping among Pacific (Alaska and East Russia) than among northern European marine populations (electronic supplementary material, figure S6 and S7). Independently of the PCA approach, for almost all LD clusters, Adriatic Sea and Iberian Peninsula freshwater populations tended to group at the opposite end of the axis than the marine populations and closely with freshwater populations from the Eastern Pacific. In contrast, the northern European freshwater populations showed a more heterogeneous pattern (electronic supplementary material, figure S6 and S7). In other words, Adriatic Sea and Iberian Peninsula freshwater populations showed more genetic parallelism than northern European freshwater populations.

## 4. Discussion

Analyses of nucleotide diversity and runs of homozygosity indicate that in contrast to northern European populations, the southern European populations have lost genetic diversity due to population bottlenecks and inbreeding and that they have experienced stronger drift resulting in higher degree of genetic differentiation. However, contrary to what we expected, southern European freshwater populations exhibited a higher degree of genetic parallelism than those from northern Europe. This suggests that the loss of genetic diversity in southern populations likely occurred after they had adapted to freshwater environments. Counterintuitively, freshwater populations in northern Europe, which have high genetic diversity and are connected to marine populations, show the lowest levels of parallelism. Below, we discuss these findings and evaluate the hypotheses that could explain these somewhat surprising results.

The observed latitudinal trend in genetic diversity is the exact opposite of what is generally expected from a recolonization of higher latitudes from southern refugia [46,47]. This is however understandable in light of the fact that many three-spined stickleback populations in the south have experienced long-term isolation [27,40,69], as indicated by our finding of higher population structure in the south than in the north. Furthermore, ROH analyses indicated that population bottlenecks and inbreeding have contributed to the lower genetic diversity in the south. For instance, the Adriatic Sea AD-KR population showed clear signs of being both bottlenecked and consanguineous [61]. While the other southern populations did not show such clear patterns, some other Adriatic Sea populations (AD-NB and AD-BOS) displayed patterns consistent with population bottlenecks and the Iberian population IP-SA showed indications of consanguinity [61].

Assuming that the likely isolation and consequent loss of access to SGV harboured by marine populations reduces the likelihood of parallel evolution (and in general of local adaptation), we expected southern European populations to display reduced levels of genetic parallelism when compared to northern European freshwater populations, which are still in contact with marine populations, and therefore, have access to their reservoir of SGV. However, our results contradict this hypothesis: for most LD clusters of global parallelism individuals from southern Europe were more likely to harbour freshwater-adapted genotypes than their northern European counterparts, and showed higher parallelism index.

This suggests that the loss of genetic diversity that characterized populations from the Iberian Peninsula and the Mediterranean basin is the result of a process that started after these populations invaded and adapted to the freshwater environment. We further suggest that the lower degree of genetic parallelism in northern than in southern Europe could be explained by differences in ongoing gene flow between northern and southern European populations. Contact between marine and freshwater populations in northern Europe provides freshwater populations with adaptive variation from a common pool of SGV, but strong gene flow can limit local adaptation by counteracting allele frequency changes driven by selection [20]. Such gene swamping could explain the reduced level of genetic parallelism in northern freshwater populations [70,71]. Furthermore, Magalhaes *et al*. [72] showed that there is an association between the extent of genetic parallelism and environmental similarities between freshwater habitats. It is therefore possible that differences in environmental homogeneity in southern and northern European freshwater populations may play a role in shaping the observed patterns of genetic parallelism. Differences in age of southern (old) and northern (young) European populations are unlikely to explain the differences in the degree of parallelism, as local adaptation from SGV can be established very rapidly [17,37,73].

For some of the LD clusters of global parallelism, there were no differences between northern European marine and freshwater populations, supporting the notion that global parallelism in three-spined stickleback populations is not as pervasive as previously thought [3], especially when there is marine-freshwater gene flow. To further confirm these results, other regions known to be associated with marine-freshwater adaptation but not exhibiting high LD (e.g. chrVII [23]) could be studied in the future. On the other hand, although Alaskan marine and freshwater populations are still in contact, they displayed more distinct patterns of freshwater parallelism than northern European marine and freshwater populations. There are at least two non-mutually exclusive explanations for this: (1) there is less gene flow between marine and freshwater populations in the Pacific than in northern Europe, and (2) Pacific freshwater populations are the result of ancient differentiation followed by secondary contact, and thus have not been independently colonized as in the rest of its distribution range [3,74].

Looking specifically at the LD clusters representing chromosomal inversions, individuals from populations in the Iberian Peninsula tended to have the haplotype associated with freshwater adaptation. In contrast, freshwater populations in northern Europe were polymorphic and many individuals carried the marine haplotype. These patterns suggest that the strength of gene flow between marine and freshwater populations may play an important role in determining the predominant inversion variant in freshwater populations. In a scenario with two alleles and antagonistic environmental effects, gene swamping is expected when *m*/*s* > *α*/∣1−*α*∣ (where *m* is the migration rate, *s* the selection coefficient and *α* the antagonistic environmental effect [20]). In southern Europe, the lack of contact between freshwater and marine populations could have facilitated selection of freshwater variants for some inversions. In contrast, in northern Europe high migration rates may be limiting the efficiency of selection. We also observed some heterogeneity in southern European freshwater populations, with some populations showing higher frequencies of freshwater adapted genotypes than others. This could be due to either geographic heterogeneity in the strength of selection or the stochastic loss of freshwater-adapted karyotypes during bottlenecks at the time of colonization. The first hypothesis seems more plausible in the case of populations with the different karyotype variants for the same inversion, while the second in the case of populations with only one inversion variant.

In conclusion, the results show that the southern European stickleback populations have reduced levels of genetic diversity and generally exhibit a higher level of genetic parallelism than their northern European contemporaries. This suggests that the high degree of genetic parallelism in the south is likely the result of the early colonization history of these populations, and that the loss of diversity occurred subsequently after the ancestral marine population was eradicated. We further suggest that the lower degree of genetic parallelism in northern than southern European populations could be a result of gene swamping, explaining the similarities between northern European marine and freshwater populations both in the genetic diversity and parallelism analyses.

## Data Availability

Raw sequence data can be found in NCBI Short Reads Archive (Bioproject PRJNA1074927, runs SRR27925730-SRR27925878). The scripts and pipelines used for the analysis of data are accessible via GitHub (https://github.com/Nopaoli/3s-Parallel-Evolution) and in Dryad (https://doi.org/10.5061/dryad.fxpnvx102) [57]. Supplementary methods, figures and tables are available in the electronic supplementary material [75].

## References

[1] Schluter D, Conte GL. 2009 Genetics and ecological speciation. Proc. Natl Acad. Sci. USA **106**, 9955-9962. (10.1073/pnas.0901264106) 19528639 PMC2702799

[2] Stern DL. 2013 The genetic causes of convergent evolution. Nat. Rev. Genet. **14**, 751-764. (10.1038/nrg3483) 24105273

[3] Fang B, Kemppainen P, Momigliano P, Feng X, Merilä J. 2020 On the causes of geographically heterogeneous parallel evolution in sticklebacks. Nat. Ecol. Evol. **4**, 1105-1115. (10.1038/s41559-020-1222-6) 32572218

[4] Endler JA. 1986 Natural selection in the wild. Princeton, NJ: Princeton University Press.

[5] MacPherson A, Nuismer SL. 2017 The probability of parallel genetic evolution from standing genetic variation. J. Evol. Biol. **30**, 326-337. (10.1111/jeb.13006) 27801996

[6] Burke MK, Liti G, Long AD. 2014 Standing genetic variation drives repeatable experimental evolution in outcrossing populations of *Saccharomyces cerevisiae*. Mol. Biol. Evol. **31**, 3228-3239. (10.1093/molbev/msu256) 25172959 PMC4245818

[7] Roesti M, Kueng B, Moser D, Berner D. 2015 The genomics of ecological vicariance in threespine stickleback fish. Nat. Commun. **6**, 8767. (10.1038/ncomms9767) 26556609 PMC4659939

[8] Reid NM et al. 2016 The genomic landscape of rapid repeated evolutionary adaptation to toxic pollution in wild fish. Science **354**, 1305-1308. (10.1126/science.aah4993) 27940876 PMC5206662

[9] Lai Y-T et al. 2019 Standing genetic variation as the predominant source for adaptation of a songbird. Proc. Natl Acad. Sci. USA **116**, 2152-2157. (10.1073/pnas.1813597116) 30659151 PMC6369788

[10] Faria R et al. 2019 Multiple chromosomal rearrangements in a hybrid zone between *Littorina saxatilis* ecotypes. Mol. Ecol. **28**, 1375-1393. (10.1111/mec.14972) 30537056 PMC6518922

[11] Van Belleghem SM, Vangestel C, De Wolf K, De Corte Z, Möst M, Rastas P, De Meester L, Hendrickx F. 2018 Evolution at two time frames: polymorphisms from an ancient singular divergence event fuel contemporary parallel evolution. PLoS Genet. **14**, e1007796. (10.1371/journal.pgen.1007796) 30422983 PMC6258555

[12] Nelson TC, Cresko WA. 2018 Ancient genomic variation underlies repeated ecological adaptation in young stickleback populations. Evol. Lett. **2**, 9-21. (10.1002/evl3.37) 30283661 PMC6121857

[13] Bailey SF, Blanquart F, Bataillon T, Kassen R. 2017 What drives parallel evolution? How population size and mutational variation contribute to repeated evolution. Bioessays **39**, 1-9. (10.1002/bies.201600176) 27859467

[14] Szendro IG, Franke J, de Visser JAGM, Krug J. 2013 Predictability of evolution depends nonmonotonically on population size. Proc. Natl Acad. Sci. USA **110**, 571-576. (10.1073/pnas.1213613110) 23267075 PMC3545761

[15] Fang B, Kemppainen P, Momigliano P, Feng X, Merilä J. 2021 Author correction: On the causes of geographically heterogeneous parallel evolution in sticklebacks. Nat. Ecol. Evol. **5**, 864. (10.1038/s41559-021-01447-7) 33888879

[16] Kemppainen P, Li Z, Rastas P, Löytynoja A, Fang B, Yang J, Guo B, Shikano T, Merilä J. 2021 Genetic population structure constrains local adaptation in sticklebacks. Mol. Ecol. **30**, 1946-1961. (10.1111/mec.15808) 33464655

[17] Cresko WA, Amores A, Wilson C, Murphy J, Currey M, Phillips P, Bell MA, Kimmel CB, Postlethwait JH. 2004 Parallel genetic basis for repeated evolution of armor loss in Alaskan threespine stickleback populations. Proc. Natl Acad. Sci. USA **101**, 6050-6055. (10.1073/pnas.0308479101) 15069186 PMC395921

[18] Orr HA. 2005 The probability of parallel evolution. Evolution **59**, 216-220. 15792240

[19] Kimura M. 1964 Diffusion models in population genetics. J. Appl. Prob. **1**, 177-232. (10.2307/3211856)

[20] Lenormand T. 2002 Gene flow and the limits to natural selection. Trends Ecol. Evol. **17**, 183-189. (10.1016/S0169-5347(02)02497-7)

[21] Yeaman S, Whitlock MC. 2011 The genetic architecture of adaptation under migration-selection balance. Evolution **65**, 1897-1911. (10.1111/j.1558-5646.2011.01269.x) 21729046

[22] Colosimo PF et al. 2005 Widespread parallel evolution in sticklebacks by repeated fixation of ectodysplasin alleles. Science **307**, 1928-1933. (10.1126/science.1107239) 15790847

[23] Jones FC et al. 2012 The genomic basis of adaptive evolution in threespine sticklebacks. Nature **484**, 55-61. (10.1038/nature10944) 22481358 PMC3322419

[24] Kakioka R, Mori S, Kokita T, Hosoki TK, Nagano AJ, Ishikawa A, Kume M, Toyoda A, Kitano J. 2020 Multiple waves of freshwater colonization of the three-spined stickleback in the Japanese Archipelago. BMC Evol. Biol. **20**, 143. (10.1186/s12862-020-01713-5) 33143638 PMC7641863

[25] Woolton RJ. 2022 A functional biology of sticklebacks. Berkeley, CA: University of California Press.

[26] Bell MA, Foster SA. 1994 Introduction to the evolutionary biology of the threespine stickleback. In The evolutionary biology of the threespine stickleback (eds MA Bell, SA Foster), pp. 1-27. Oxford, UK: Oxford University Press.

[27] Mäkinen HS, Merilä J. 2008 Mitochondrial DNA phylogeography of the three-spined stickleback (Gasterosteus aculeatus) in Europe-evidence for multiple glacial refugia. Mol. Phylogenet. Evol. **46**, 167-182. (10.1016/j.ympev.2007.06.011) 17716925

[28] Sychevskaya YK, Grechina NI. 1981 Fossil sticklebacks of the genus Gasterosteus from the Neogene of the Soviet Far East. Paleontol. J **1**, 71-80.

[29] Bell MA, Stewart JD, Park PJ. 2009 The world's oldest fossil threespine stickleback fish. Copeia **2009**, 256-265. (10.1643/CG-08-059)

[30] Fang B, Merilä J, Ribeiro F, Alexandre CM, Momigliano P. 2018 Worldwide phylogeny of three-spined sticklebacks. Mol. Phylogenet. Evol **127**, 613-625. (10.1016/j.ympev.2018.06.008) 29906607

[31] McKinnon JS, Rundle HD. 2002 Speciation in nature: the threespine stickleback model systems. Trends Ecol. Evol. **17**, 480-488. (10.1016/S0169-5347(02)02579-X)

[32] Mäkinen HS, Cano JM, Merilä J. 2006 Genetic relationships among marine and freshwater populations of the European three-spined stickleback (*Gasterosteus aculeatus*) revealed by microsatellites. Mol. Ecol. **15**, 1519-1534. (10.1111/j.1365-294X.2006.02871.x) 16629808

[33] Mazzarella AB, Boessenkool S, Østbye K, Vøllestad LA, Trucchi E. 2016 Genomic signatures of the plateless phenotype in the threespine stickleback. Ecol. Evol. **6**, 3161-3173. (10.1002/ece3.2072) 27096077 PMC4829042

[34] Myhre F, Klepaker T. 2009 Body armour and lateral-plate reduction in freshwater three-spined stickleback *Gasterosteus aculeatus*: adaptations to a different buoyancy regime? J. Fish Biol. **75**, 2062-2074. (10.1111/j.1095-8649.2009.02404.x) 20738672

[35] McPhail JD, Hay DE. 1983 Differences in male courtship in freshwater and marine sticklebacks (*Gasterosteus aculeatus*). Can. J. Zool. **61**, 292-297. (10.1139/z83-039)

[36] Leinonen T, McCairns RJS, Herczeg G, Merilä J. 2012 Multiple evolutionary pathways to decreased lateral plate coverage in freshwater threespine sticklebacks. Evolution **66**, 3866-3875. (10.1111/j.1558-5646.2012.01724.x) 23206143

[37] Terekhanova NV, Logacheva MD, Penin AA, Neretina TV, Barmintseva AE, Bazykin GA, Kondrashov AS, Mugue NS. 2014 Fast evolution from precast bricks: genomics of young freshwater populations of threespine stickleback *Gasterosteus aculeatus*. PLoS Genet. **10**, e1004696. (10.1371/journal.pgen.1004696) 25299485 PMC4191950

[38] Liu S, Hansen MM, Jacobsen MW. 2016 Region-wide and ecotype-specific differences in demographic histories of threespine stickleback populations, estimated from whole genome sequences. Mol. Ecol. **25**, 5187-5202. (10.1111/mec.13827) 27569902

[39] Fang B, Kemppainen P, Momigliano P, Merilä J. 2021 Population structure limits parallel evolution in sticklebacks. Mol. Biol. Evol. **38**, 4205-4221. (10.1093/molbev/msab144) 33956140 PMC8476136

[40] DeFaveri J, Zanella LN, Zanella D, Mrakovčić M, Merilä J. 2012 Phylogeography of isolated freshwater three-spined stickleback *Gasterosteus aculeatus* populations in the Adriatic Sea basin. J. Fish Biol. **80**, 61-85. (10.1111/j.1095-8649.2011.03147.x) 22220890

[41] Fang B, Merilä J, Matschiner M, Momigliano P. 2020 Estimating uncertainty in divergence times among three-spined stickleback clades using the multispecies coalescent. Mol. Phylogenet. Evol. **142**, 106646. (10.1016/j.ympev.2019.106646) 31634562

[42] Lucek K, Seehausen O. 2015 Distinctive insular forms of threespine stickleback (*Gasterosteus aculeatus*) from western Mediterranean islands. Conserv. Genet. **16**, 1319-1333. (10.1007/s10592-015-0742-0)

[43] Sanz N, Araguas RM, Vidal O, Viñas J. 2015 Glacial refuges for three-spined stickleback in the Iberian Peninsula: mitochondrial DNA phylogeography. Freshw. Biol. **60**, 1794-1809. (10.1111/fwb.12611)

[44] Ramachandran S, Deshpande O, Roseman CC, Rosenberg NA, Feldman MW, Cavalli-Sforza LL. 2005 Support from the relationship of genetic and geographic distance in human populations for a serial founder effect originating in Africa. Proc. Natl Acad. Sci. USA **102**, 15 942-15 947. (10.1073/pnas.0507611102) 16243969 PMC1276087

[45] Dahms C, Kemppainen P, Zanella LN, Zanella D, Carosi A, Merilä J, Momigliano P. 2022 Cast away in the Adriatic: low degree of parallel genetic differentiation in three-spined sticklebacks. Mol. Ecol. **31**, 1234-1253. (10.1111/mec.16295) 34843145

[46] Hewitt GM. 2008 Post-glacial re-colonization of European biota. Biol. J. Linn. Soc. Lond. **68**, 87-112. (10.1111/j.1095-8312.1999.tb01160.x)

[47] Lumibao CY, Hoban SM, McLachlan J. 2017 Ice ages leave genetic diversity ‘hotspots’ in Europe but not in Eastern North America. Ecol. Lett. **20**, 1459-1468. (10.1111/ele.12853) 28942617

[48] Poizat G, Rosecchi E, Crivelli AJ. 2002 Life-history variation within a three-spined stickleback population in the Camargue. J. Fish Biol. **60**, 1296-1307.

[49] Cano JM, Mäkinen HS, Leinonen T, Freyhof J, Merilä J. 2008 Extreme neutral genetic and morphological divergence supports classification of Adriatic three-spined stickleback (*Gasterosteus aculeatus*) populations as distinct conservation units. Biol. Conserv. **141**, 1055-1066. (10.1016/j.biocon.2008.01.015)

[50] Miller MR, Dunham JP, Amores A, Cresko WA, Johnson EA. 2007 Rapid and cost-effective polymorphism identification and genotyping using restriction site associated DNA (RAD) markers. Genome Res. **17**, 240-248. (10.1101/gr.5681207) 17189378 PMC1781356

[51] Baird NA, Etter PD, Atwood TS, Currey MC, Shiver AL, Lewis ZA, Selker EU, Cresko WA, Johnson EA. 2008 Rapid SNP discovery and genetic mapping using sequenced RAD markers. PLoS One **3**, e3376. (10.1371/journal.pone.0003376) 18852878 PMC2557064

[52] Catchen J, Hohenlohe PA, Bassham S, Amores A, Cresko WA. 2013 Stacks: an analysis tool set for population genomics. Mol. Ecol. **22**, 3124-3140. (10.1111/mec.12354) 23701397 PMC3936987

[53] Hubbard T et al. 2005 Ensembl 2005. Nucleic Acids Res. **33**, D447-D453. (10.1093/nar/gki138) 15608235 PMC540092

[54] Li H, Durbin R. 2010 Fast and accurate long-read alignment with Burrows-Wheeler transform. Bioinformatics **26**, 589-595. (10.1093/bioinformatics/btp698) 20080505 PMC2828108

[55] Li H, Handsaker B, Wysoker A, Fennell T, Ruan J, Homer N, Marth G, Abecasis G, Durbin R. 2009 The sequence alignment/map format and SAMtools. Bioinformatics **25**, 2078-2079. (10.1093/bioinformatics/btp352) 19505943 PMC2723002

[56] McKenna A et al. 2010 The Genome Analysis Toolkit: a MapReduce framework for analyzing next-generation DNA sequencing data. Genome Res. **20**, 1297-1303. (10.1101/gr.107524.110) 20644199 PMC2928508

[57] Coll-Costa C, Dahms C, Kemppainen P, Alexandre CM, Ribeiro F, Zanella D, Zanella L, Merilä J, Momigliano P. 2024 Data from: Parallel evolution despite low genetic diversity in three-spined sticklebacks. *Dryad Digital Repository*. (10.5061/dryad.fxpnvx102) PMC11003780 38593844

[58] Nei M, Li W-H. 1979 Mathematical model for studying genetic variation in terms of restriction endonucleases. Proc. Natl Acad. Sci. USA **76**, 5269-5273. (10.1073/pnas.76.10.5269) 291943 PMC413122

[59] Momigliano P, Florin A-B, Merilä J. 2021 Biases in demographic modelling affect our understanding of recent divergence. Mol. Biol. Evol. **38**, 2967-2985. 33624816 10.1093/molbev/msab047 PMC8233503

[60] Narasimhan V, Danecek P, Scally A, Xue Y, Tyler-Smith C, Durbin R. 2016 BCFtools/RoH: a hidden Markov model approach for detecting autozygosity from next-generation sequencing data. Bioinformatics **32**, 1749-1751. (10.1093/bioinformatics/btw044) 26826718 PMC4892413

[61] Ceballos FC, Joshi PK, Clark DW, Ramsay M, Wilson JF. 2018 Runs of homozygosity: windows into population history and trait architecture. Nat. Rev. Genet. **19**, 220. (10.1038/nrg.2017.109) 29335644

[62] Keenan K, McGinnity P, Cross TF, Crozier WW, Prodöhl PA. 2013 diveRsity: an R package for the estimation and exploration of population genetics parameters and their associated errors. Methods Ecol. Evol. **4**, 782-788. (10.1111/2041-210X.12067)

[63] Purcell S et al. 2007 PLINK: a tool set for whole-genome association and population-based linkage analyses. Am. J. Hum. Genet. **81**, 559-575. 17701901 10.1086/519795 PMC1950838

[64] Liu X, Fu Y-X. 2015 Exploring population size changes using SNP frequency spectra. Nat. Genet. **47**, 555-559. (10.1038/ng.3254) 25848749 PMC4414822

[65] Kemppainen P, Knight CG, Sarma DK, Hlaing T, Prakash A, Maung Maung YN, Somboon P, Mahanta J, Walton C. 2015 Linkage disequilibrium network analysis (LDna) gives a global view of chromosomal inversions, local adaptation and geographic structure. Mol. Ecol. Resour. **15**, 1031-1045. (10.1111/1755-0998.12369) 25573196 PMC4681347

[66] Meisner J, Albrechtsen A. 2018 Inferring population structure and admixture proportions in low-depth NGS data. Genetics **210**, 719. (10.1534/genetics.118.301336) 30131346 PMC6216594

[67] Eddelbuettel D, Francois R. 2011 Rcpp: seamless R and C++ integration. J. Stat. Softw. **40**, 1-18.

[68] James DA, Venables WN, Ripley BD. 1996 Modern applied statistics with S-PLUS. Technometrics **38**, 77.

[69] Zanella LN, DeFaveri J, Zanella D, Merilä J, Šanda R, Mrakovčić M. 2015 Does predation drive morphological differentiation among Adriatic populations of the three-spined stickleback? Biol. J. Linn. Soc. Lond. **115**, 219-240. (10.1111/bij.12491)

[70] Marques DA, Lucek K, Meier JI, Mwaiko S, Wagner CE, Excoffier L, Seehausen O. 2016 Genomics of rapid incipient speciation in sympatric threespine stickleback. PLoS Genet. **12**, e1005887. (10.1371/journal.pgen.1005887) 26925837 PMC4771382

[71] Stuart YE et al. 2017 Contrasting effects of environment and genetics generate a continuum of parallel evolution. Nat Ecol Evol **1**, 158. (10.1038/s41559-017-0158) 28812631

[72] Magalhaes IS, Whiting JR, D'Agostino D, Hohenlohe PA, Mahmud M, Bell MA, Skúlason S, MacColl ADC. 2021 Intercontinental genomic parallelism in multiple three-spined stickleback adaptive radiations. Nat Ecol Evol **5**, 251-261. (10.1038/s41559-020-01341-8) 33257817 PMC7858233

[73] Lescak EA, Bassham SL, Catchen J, Gelmond O, Sherbick ML, von Hippel FA, Cresko WA. 2015 Evolution of stickleback in 50 years on earthquake-uplifted islands. Proc. Natl Acad. Sci. USA **112**, E7204-E7212. (10.1073/pnas.1512020112) 26668399 PMC4702987

[74] Bierne N, Gagnaire PA, David P. 2013 The geography of introgression in a patchy environment and the thorn in the side of ecological speciation. Curr. Zool. **59**, 72-86. (10.1093/czoolo/59.1.72)

[75] Coll-Costa C, Dahms C, Kemppainen P, Alexandre CM, Ribeiro F, Zanella D, Zanella L, Merilä J, Momigliano P. 2024 Parallel evolution despite low genetic diversity in three-spined sticklebacks. *Figshare*. (10.6084/m9.figshare.c.7132044) PMC11003780 38593844

